# A novel strategy for production of liraglutide precursor peptide and development of a new long-acting incretin mimic

**DOI:** 10.1371/journal.pone.0266833

**Published:** 2022-05-02

**Authors:** Samaneh Ahmadi, Mohammad Bagher Shahsavani, Zohreh Tavaf, Rawayh Muslim Albaghlany, Ashutosh Kumar, Ali Akbar Moosavi-Movahedi, Reza Yousefi

**Affiliations:** 1 Department of Biology, Protein Chemistry Laboratory (PCL), College of Sciences, Shiraz University, Shiraz, Iran; 2 Department of Biosciences and Bioengineering, IIT Bombay, Powai, Mumbai, India; 3 Institute of Biochemistry and Biophysics (IBB), University of Tehran, Tehran, Iran; Bhabha Atomic Research Centre, INDIA

## Abstract

Nowadays, a small number of incretin mimics are used to treat type 2 diabetes mellitus (T2DM) due to their longer half-life. The present study aimed to introduce a novel method for producing the liraglutide precursor peptide (LPP) and developing a potentially new incretin mimic. Here, human αB-crystallin (αB-Cry) was ligated to the LPP at the gene level, and the gene construct was expressed in *Escherichia coli* with a relatively good efficiency. The hybrid protein (αB-lir) was then purified by a precipitation method followed by anion exchange chromatography. After that, the peptide was released from the carrier protein by a chemical cleavage method yielding about 70%. The LPP was then purified by gel filtration chromatography, and HPLC estimated its purity to be about 98%. Also, the molecular mass of the purified peptide was finally confirmed by mass spectroscopy analysis. Assessment of the secondary structures suggested a dominant α-helical structure for the LPP and a β-sheet rich structure for the hybrid protein. The subcutaneous injection of the LPP and the αB-lir hybrid protein significantly reduced the blood sugar levels in healthy and diabetic mice and stimulated insulin secretion. Also, the hybrid protein exerts its bioactivities more effectively than the LPP over a relatively longer period of time. The results of this study suggested a novel method for the easy and cost-effective production of the LPP and introduced a new long-acting incretin mimic that can be potentially used for the treatment of T2DM patients.

## 1. Introduction

The recent evidence suggested that insulin secretion is reduced by about 50% in patients with type 2 diabetes mellitus (T2DM) [1]. Previous investigations on diabetes have highlighted the critical role of incretin hormones in maintaining glucose control as they are estimated for approximately 50–70% of the total insulin secretion at a glucose plasma level >4 mM [2, 3]. Glucagon-like peptide-1 (GLP-1), as an incretin hormone, produces after posttranslational processing of the proglucagon gene product by a prohormone convertase (PC)1/3, expressing in the enteroendocrine L-cells [4, 5]. GLP-1 has been well known for its prominent activity of glucose-dependent insulin secretion in the pancreatic β-cells. Other significant biological functions of this peptide hormone are listed as inducing expression of the proinsulin gene, playing a role in the inhibition of glucagon release, stimulating pancreatic β-cells proliferation, inducing neogenesis, inhibiting β-cells apoptosis, and creating a significant delay in the gastric emptying [6, 7]. The GLP-1 secretion after nutrient consumption ceases when the blood glucose returns to the normal level, effectively avoiding the hypoglycemia symptoms, which is one of the main side effects associated with the most widely used modalities of DM treatment [8, 9]. However, the majority of GLP-1 in the bloodstream is rapidly degraded by dipeptidyl peptidase-IV (DPP-IV) [10], as well as a small quantity can also be cleaved by neutral endopeptidase (NEP) 24.11, at the multiple positions [11]. Thus, the native GLP-1 cannot be effectively applied for therapeutic purposes due to the fast and ubiquitous degradation.

The two types of clinically approved drugs have emerged based on the physiological action of GLP-1 [12]. The first group is GLP-1 receptor agonists (GLP-1RAs), such as liraglutide, dulaglutide, albiglutide, exenatide, and semaglutide for injectable usages [13, 14]. The second group is the orally active DPP-IV inhibitors, including sitagliptin, saxagliptin, linagliptin, vildagliptin, which currently received also much attention in the treatment of Covid-19, as well [15–17]. The GLP-1RAs that bind to the GLP-1 receptor (GLP-1R) with a similar affinity can be classified into two categories; the first one is human GLP-1 analogues with a half-life that exceeds the native GLP-1. The second class of the analogues is produced based on the reptilian peptide exendin-4 with 53% of sequence homology to GLP-1 [18].

Liraglutide is the first once-daily long-acting analogue with 97% identity to the naturally occurring human GLP-1, containing a palmitoyl chain at Lys^26^ through a γ-glutamyl spacer along with a Lys to Arg substitution at position 34, and it has been constructed for the treatment of diabetes and obesity [19]. Based on the recent evidences, due to its anti-inflammatory properties, liraglutide has also been suggested as an adjunctive treatment for Covid-19 patients [15, 20]. The attachment of liraglutide to palmitate facilitates its binding to human serum albumin, preventing its fast renal clearance and shielding the specific DPP-IV cleavage site on the primary structure of this peptide medicine [21]. These changes finally increase its half-life in the bloodstream to 13 hours. Recent evidences suggested that liraglutide improves glycemic control [22], reduces body weight [23], displays a favourable effect on blood pressure [24, 25], decreases cardiovascular (CV) risk [26], and prevents neurodegenerative processes in the mouse model of Alzheimer’s disease [27].

The binding capacity of serum albumin to liraglutide, also acyl-mediated self-assembling, and heptamerization of this anti-diabetic medicine are highly important for its biological action profile [2]. However, the acylation strategy seems to be failed in patients with severe DM, because results of the recent studies suggested that excessive glycation of human serum albumin in these patients reduces to the large extent its ability for binding to the liraglutide medicine [28]. For this reason, finding a new solution to overcome this critical medical challenge is one of the urgent and essential needs of the medical community. Additionally, small peptides are difficult to obtain by conventional bioexpression and purification systems due to their high susceptibility to fast degradation. Generally, conjugating peptides with an appropriate partner protein significantly improves the level of expression and their pharmacokinetic profiles [13, 29–32]. Therefore, we developed a novel strategy of liraglutide precursor peptide (LPP) production via forming a fusion construct using human αB-crystallin (αB-Cry), as the partner protein (αB-lir). Human αB-Cry is a highly beneficial protein partner because of its several advantages, including being a molecular chaperone, its intrinsic ability to form large oligomers, facilitating its purification by size exclusion approaches, and prevention of the proteolytic degradation by the forming inclusion bodies in the bacterial expression system [33]. In this study, we expressed αB-lir fusion protein in *Escherichia coli* (*E*.*coli*). After successful purification of the hybrid protein and obtaining the final liraglutide peptide, we examined their structure, bioactivity, and glucoregulatory effects. Also, the structure and function of αB-lir was extensively examined with the aim to introduce a novel GLP-1RA for the possible application in the treatment regimen of T2DM.

## 2. Materials and methods

### 2.1. Materials

1-anilino-8-naphthalene sulfonic acid (ANS), cyanogen bromide (CNBr), kanamycin, isopropyl β-D-1 thiogalactopyranoside (IPTG), β-mercaptoethanol (β-ME), and other chemicals were purchased from Sigma. The dialysis tubes with molecular weight cut-off (MWCO) of 2 and 12 kDa were purchased from Spectrum Scientific Company. The gel filtration media were purchased from GE Healthcare and Qiagen. Insulin (INS) ELISA kit was purchased from Shanghai Crystal day Biotech Co., Ltd.

### 2.2. Methods

#### 2.2.1. Construction of pET28b(+) containing αB-lir hybrid gene

The expression construct consists of a human αB-Cry gene (*CRYAB*) that is linked to the LPP sequence. The LPP sequence was obtained from PubChem online database with the PubChem CID (16134956). The primary structure of LPP is shown in S1 Fig. The gene coding αB-lir was inserted into an appropriate expression vector via the NcoI and XhoI restriction sites. Also, a methionine residue was added to the carboxy terminus of the partner protein (αB-Cry), providing a specific CNBr cleavage site. Methionine 68 and proline 130 were mutated with isoleucine and valine, respectively. These substitutions provide resistance against cleavage by CNBr and formic acid. The lac operator has also been applied to overexpress the αB-lir. Aimed at possible use in the purification process, a six histidine residues (His-tag) was also inserted at the N-terminal of the αB-lir hybrid protein. The construct was chemically synthesized and then cloned into pET28b(+). Finally, the gene sequence of the construct was confirmed by DNA sequencing using T7 promoter primers.

#### 2.2.2. Expression of the αB-lir in the bacterial host system

The plasmid was transformed into *E*. *coli* BL21 (DE3) and a single colony was selected to the culture in 10 mL Luria-Bertani (LB) broth medium with an appropriate antibiotic (kanamycin 50 μg/mL) and constant shaking at 37°C. The overnight culture was transferred to a 1000 mL culture medium supplemented with kanamycin and incubated at the same conditions until the optical density at 600 nm reached 0.7. Then, IPTG at a final concentration of 0.25 mM was added to induce expression of the recombinant hybrid protein (αB-lir). The cells were harvested by centrifugation at 7000 ×g for 7 min. Then, the bacterial cells were resuspended in the lysis buffer (25 mM Tris, pH 8.0, containing 7 M urea) and incubated for 20 min at 37°C. The crude extract was then centrifuged with 9000 ×g for 50 min at 4°C, and the supernatant was then collected. The protein expression was analyzed using SDS-PAGE, and the protein bands were visualized by staining the gel using Coomassie Brilliant Blue (CBB) dye [34].

#### 2.2.3. Purification of the αB-lir hybrid protein

Initially, the supernatant of cell lysate was dialyzed against an appropriate volume of 25 mM Tris, pH 8.0, with the aim to reduce the final concentration of urea to 2.5 M. We observed experimentally that the recombinant αB-lir could precipitate in the Tris buffer containing 2.5 M urea. Using this precolumn purification approach, most of the protein contaminants were removed. After performing the dialysis for 4 hours, the αB-lir was precipitated, which then collected and dissolved in the same buffer containing 4 M urea. The protein solution was also subjected to a high-speed centrifugation, and the obtained supernatant was applied to the DEAE-cellulose column (7 cm × 2 cm), which was pre-equilibrated with the same buffer. The majority of αB-lir protein was eluted as the flow throw when the flow rate was fixed at 1 mL/min. The protein contaminants (the bound proteins) were also removed from the column by passing 1 M NaCl in the same buffer. This procedure was repeated twice in order to get a highly pure sample of the αB-lir hybrid protein. Finally, the αB-lir hybrid protein was subjected to the extensive dialysis against double distilled water to remove urea. The dialyzed protein solution was lyophilized using a freeze dryer (Alpha 2–4 LSCbasic, Martin Christ company) and kept at -20°C. The SDS-PAGE analysis (gel 12%) was also performed to assess the purity of the final αB-lir hybrid protein [34].

#### 2.2.4. Cleavage of the αB-lir and purification of the LPP

The CNBr is an inorganic compound commonly used to hydrolyze peptide bonds after methionine residue in protein/peptide sequence [35, 36]. The purified αB-lir protein was directly solubilized in formic acid (70% v/v) at a fixed concentration of 20 mg/mL. The CNBr solution was prepared in the same buffer with a 100-fold molar excess to the αB-lir solution (weight ratio of 1:1). The mixture was incubated at room temperature in dark conditions for 24 hours. Then, the sample was dialyzed overnight against double distilled water by an appropriate dialysis tube (2 kDa molecular weight cut-off) to remove CNBr and formic acid from the protein/peptide environment. Finally, the mixture (αB-lir, αB-Cry, LPP) was lyophilized for further purification. SDS-PAGE analysis (gel 18%) was used to elucidate the performance of αB-lir digestion.

The gel filtration chromatography on Sephadex G-50 column (80 cm × 1cm) was applied to separate the LPP from the other products. The protein solution (100 mg/mL) was then dissolved in the glacial acetic acid (20%) and subsequently loaded onto the Sephadex G-50 column equilibrated with the same buffer. The flow rate and fraction sizes were 0.2 mL/min and 2 mL, respectively. The highly pure LPP sample was gained by repeating this procedure twice. The purity of the LPP was further examined by the RP-HPLC column (ProntoSIL 200-5-C18, 250 × 4.6 mm, Apex Scientific) equipped with a UV detector (Smartline 2500, KNAUER). The peaks were observed at a flow rate of 1 mL/min at 214 nm with a linear gradient of acetonitrile (0–60%) for 15 min at a constant temperature of 25ºC [33].

#### 2.2.5. Matrix-assisted laser desorption ionization (MALDI) mass spectrometry

The mass spectra of the purified peptide were recorded on Autoflex III TOF/TOF MS (MALDI-TOF mass spectrometer, Bruker Daltonics Co.). Briefly, 2 μl of 5 μM peptide solution was mixed with 2 μl of matrix solution (α- cyano-4-hydroxy cinnamic acid). External calibration was performed with a mixture of protein standards (5–20 kDa). For HRLCMS analysis, 1μL of the standard samples and a 2 μL of test peptide sample was injected in Q-Exactive Plus Orbitrap Mass Spectrometer. The samples were run in 0.1% formic acid in water, and direct masses were observed. The chromatograms were recorded and analyzed using the X-calibur (Thermo fisher) software. Scan range was searched in the range of 1000 to 3000 m/z.

#### 2.2.6. Fourier transform infrared spectroscopy (FTIR) analysis and Raman measurements

FTIR spectra of the solid-state of the pure LPP, αB-lir, and human αB-Cry (as Ctrl sample) were measured at 25°C with a Bruker ATR-FTIR spectrophotometer (Tensor II, Germany). The spectra were recorded from 1720 to 1580 cm^-1^ using a resolution of 3 cm^-1^ and an accumulation of 64 scans. The secondary structural content was assessed by curve fitting of the amide I region (1700–1620 cm^-1^) using an appropriate curve-fitting program (GRAMS/AI^™^ Spectroscopy Software v9.2). Deconvolution analysis was carried out with the Gaussian function [37, 38].

The LabRAM HR Evolution Raman (Horiba, Japan) equipped with a confocal microscope was used for Raman spectroscopy analyses. The Raman signals were recorded in a spectral range of 1800–400 cm^-1^ with an acquisition time of 15s with the accumulation of 5 using a 532 nm green laser excitation (600 g/mm grating), with a 50x objective magnification (numerical aperture = 0.5) for focusing and collection of Raman-scattered light. Curve fitting and estimation of the secondary structural content in the amide I region (1700–1620 cm^-1^) were carried out using an appropriate curve-fitting program (GRAMS/AI^™^ Spectroscopy Software v9.2). Deconvolution analysis was carried out with the Gaussian function [38, 39].

#### 2.2.7. Circular dichroism (CD) measurement

The CD measurements were performed on a Jasco J-720 spectropolarimeter from 250 to 190 nm in a 0.1 cm path length cell at 22ºC. The LPP and αB-lir/αB-Cry were prepared in 10 mM sodium phosphate buffer (pH 8.1) and in 100 mM sodium acetate buffer (pH 5.2), respectively [40, 41]. The concentration of both samples was fixed at 0.2 mg/mL. The secondary structure analyses were predicted using the DICHROWEB server [42, 43].

#### 2.2.8. The surface hydrophobicity assessment

In order to compare the surface hydrophobicity of the αB-lir and human αB-Cry, ANS binding analysis was done (Agilent fluorescence spectrophotometer, Varian Cary Eclipse, USA). The αB-lir/αB-Cry (0.15 mg/mL) was dissolved in 50 mM sodium phosphate buffer pH 7.4 in the presence of ANS (100 μM) and incubated for 30 min in the dark condition. The excitation wavelength was set at 365 nm, and the emission spectra were collected at the wavelength range of 400–600 nm [44]. The fluorescence spectra were measured at three different temperatures as 27, 37, 47ºC.

#### 2.2.9. Dynamic light scattering (DLS) measurement

In order to investigate the oligomerization state of the αB-lir at different temperatures (27, 37, 47ºC), DLS analysis was performed by nanoparticle analyzer SZ-100 (Horiba Ltd. Japan). The αB-lir was prepared in phosphate buffer pH 7.4 at a fixed concentration of 1 mg/mL. A 173º scattering angle and a laser beam of 532 nm were used. The protein analysis of the inbuilt software (S-Z 100 for windows) was used to calculate the size distribution of protein particles [45].

### 2.3. Intraperitoneal glucose tolerance tests (IPGTT) on healthy and diabetic mice

The Intraperitoneal glucose tolerance tests (IPGTT) was conducted on 6-weeks old BALB/c male mice (weighting 20–27 g) that had fasted for six hours according to the protocol reported previously [46, 47]. Briefly, the mice were subjected to 12/12 hours of light/dark cycle and were given free access to standard food and water. They were also randomly divided into control and test groups (n = 6), while each group was administered 1.5 mg/g body weight glucose into the intraperitoneal cavity. The treatment group received a single dose injection of the LPP (dissolved in 10 mM Phosphate-Buffered Saline (PBS) at pH 7.4) via intraperitoneal (IP) injection at a concentration of 200 μg/Kg body weight [47, 48] and the control group received an equivalent amount of PBS. Before the injection, the glucose concentration in the collected blood samples from the cut tail vein was measured for 30 min. After injection, the blood glucose was measured for each mice at 0, 10, 20, 30, 40, 50, 60, 90, 120 minutes with a GDH blood glucose meter (TD-4277) [47, 49]. The blood-glucose-lowering activity of αB-lir (1.4 mg/Kg body weight) was also tested in mice, using the same procedure with the exception that the protein injection was done 24 hours before the test. We followed a similar method that reported previously to dissolve the hybrid protein in PBS [47–49].

Diabetes was induced in mice using streptozotocin (STZ), a compound with preferential toxicity toward pancreatic β-cells [50]. Two groups of 6-week-old mice (n = 6) were chosen for an intraperitoneal injection with a 30 mg/Kg body weight dose of streptozotocin (STZ) dissolved in citrate buffer (50 mM) for two weeks. The STZ-induced diabetic mice were selected and randomly divided into two groups. The blood glucose level was measured as described above. Also, in the case of the αB-lir a similar procedure as mentioned above was applied.

#### 2.3.1. The assay of insulin secretion after stimulation by the LPP and αB-lir hybrid protein

The measuring blood glucose level alone is not sufficient to evaluate the state of sugar metabolism. Therefore, we decided to assay the blood insulin after injection of the incretin mimics. The overall procedure and steps for the insulin assay were similar to those used for the assay of blood sugar levels. When the concentration of blood glucose reached its maximum, the insulin concentration was measured using a suitable insulin ELISA kit (Shanghai Crystal day Biotech Co.) based on the protocol which has been reported in previous publications [51]. The insulin concentration was calculated using a standard curve.

#### 2.3.2. Ethics statement

In this study, the mice were purchased from Razi Vaccine and Serum Research Institute (Iran). All biological activity analyses followed the ethical guidelines for animal experiments were described and approved by the committee for experiments with laboratory animals of the National Research Ethics Committee [31].

### 2.4. The statistical analysis

The data were statistically analyzed by one-way ANOVA with Bonferroni’s test and Multiple T-test, using GraphPad Prism 9.2. The P-value <0.05 was considered significant among the different groups.

## 3. Results

### 3.1. Designing the appropriate gene construct, expression and purification of the αB-lir hybrid protein

**Fig 1:**
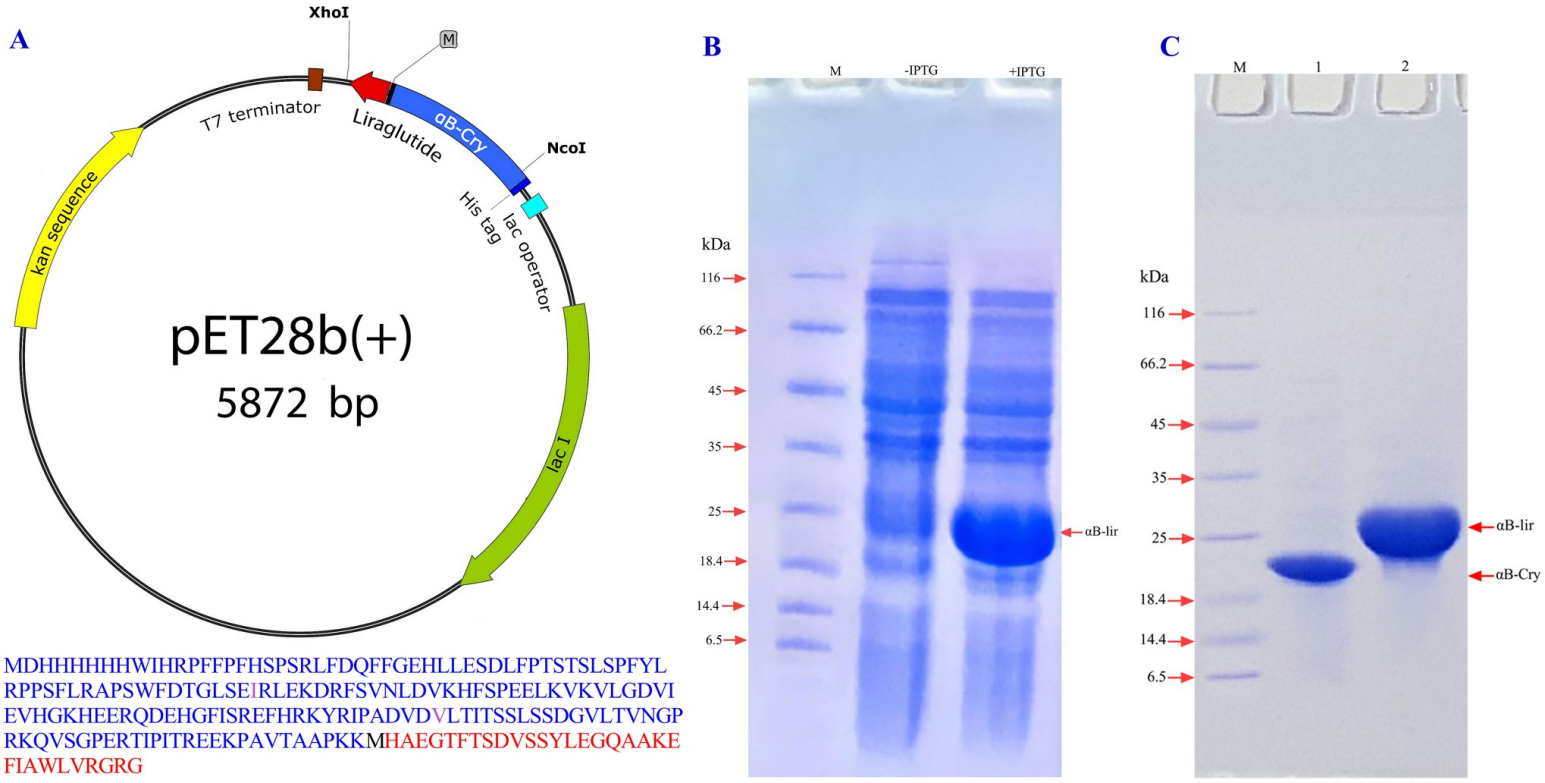
From designing the gene construct to the expression and purification of αB-lir hybrid protein. **A**) pET28b(+) vectors containing the hybrid gene (αB-lir) are demonstrated. The positions of NcoI and XhoI restriction sites are also indicated. The translation frame on this vector shows the amino acid sequence of αB-lir hybrid protein. The human αB-Cry gene is portrayed in purple, and the primary structure of the LPP is shown as red. Also, M stands for the methionine residue that provides a CNBr specific cleavage site. In the primary structure of the human αB-Cry gene, methionine 68 and proline 130 are substituted with isoleucine and valine residues, respectively. **B**) The expression of αB-lir hybrid protein was assessed by SDS-PAGE analysis (gel 12%). The αB-lir expression in the absence and presence of 0.25 mM IPTG is indicated, and M shows the protein mass markers. **C**) The αB-lir was purified using the precipitation method followed by a DEAE column. Then, the pure αB-lir was analyzed on a reducing SDS-PAGE (gel 12%). Lanes **1** and **2** stand for human αB-Cry and αB-lir hybrid protein, respectively. Also, M indicated the protein mass marker.

In order to produce the LPP in appropriate quantities, it was fused to the human αB-crystallin at the gene level (Fig 1). The aim was to increase the expression yields of this anti-diabetic peptide in *Escherichia coli* host cells. In the primary structure, the liraglutide lacks methionine residue, but human αB-crystallin contains only one methionine at position 68, which challenges the separation of the drug peptide from the carrier protein by the chemical digestion in the presence of cyanogen bromide; thus, this residue (Met 68) was substituted with isoleucine.

In order to separate the LPP from the partner protein, the chemical cleavage method by cyanogen bromide at the boundary methionine was used. Also, since the chemical cleavage was done in the acidic condition, and under such conditions, cleaving of the peptide bond also occurs at the aspartic acid-proline (DP) boundary position, the proline residue at position 130 of human αB-crystallin was replaced with valine (V). Moreover, a methionine residue was added to the carboxy terminus of human αB-crystallin. The presence of the methionine at the boundary of human αB-crystallin and LPP facilitates releasing of the therapeutic peptide from the carrier protein by cyanogen bromide chemical cleavage (Fig 1A). Finally, the cDNA sequence of this protein was cloned using NcoI and XhoI restriction enzymes in the pET28b(+) vector, and the precision of its nucleotide sequence was confirmed by DNA sequencing.

After cloning of the gene construct into *Escherichia coli* BL21 (DE3), the transformed bacteria were cultured in the LB medium at 37°C, and the expression of the hybrid protein was induced by IPTG (0.25 mM), while the incubation was continued for 12 hours. After digestion of the bacterial pellet, the expression of the hybrid protein was assessed by SDS-PAGE (Fig 1B). The results indicated a significant expression of the αB-lir hybrid protein with high efficacy. A two-step purification procedure, including precipitation in Tris buffer, was subsequently followed by ion-exchange chromatography on diethylaminoethyl (DEAE) cellulose matrix, was used to purify αB-lir hybrid protein (Fig 1C). Using this purification approach, approximately 125 mg of αB-lir was obtained from one liter of LB culture medium. The SDS-PAGE analysis was used to check the purity of the hybrid protein. On the SDS-PAGE gel, the purified hybrid protein indicated a molecular mass of about 23 kDa, which corresponds to the calculated molecular weight according to its primary structure (Fig 1C). The results obtained by SDS-PAGE analysis also confirm that the obtained protein (αB-lir) is of high purity (more than 97%).

### 3.2. Chemical cleavage of the hybrid protein (αB-lir) and purification of the LPP

**Fig 2:**
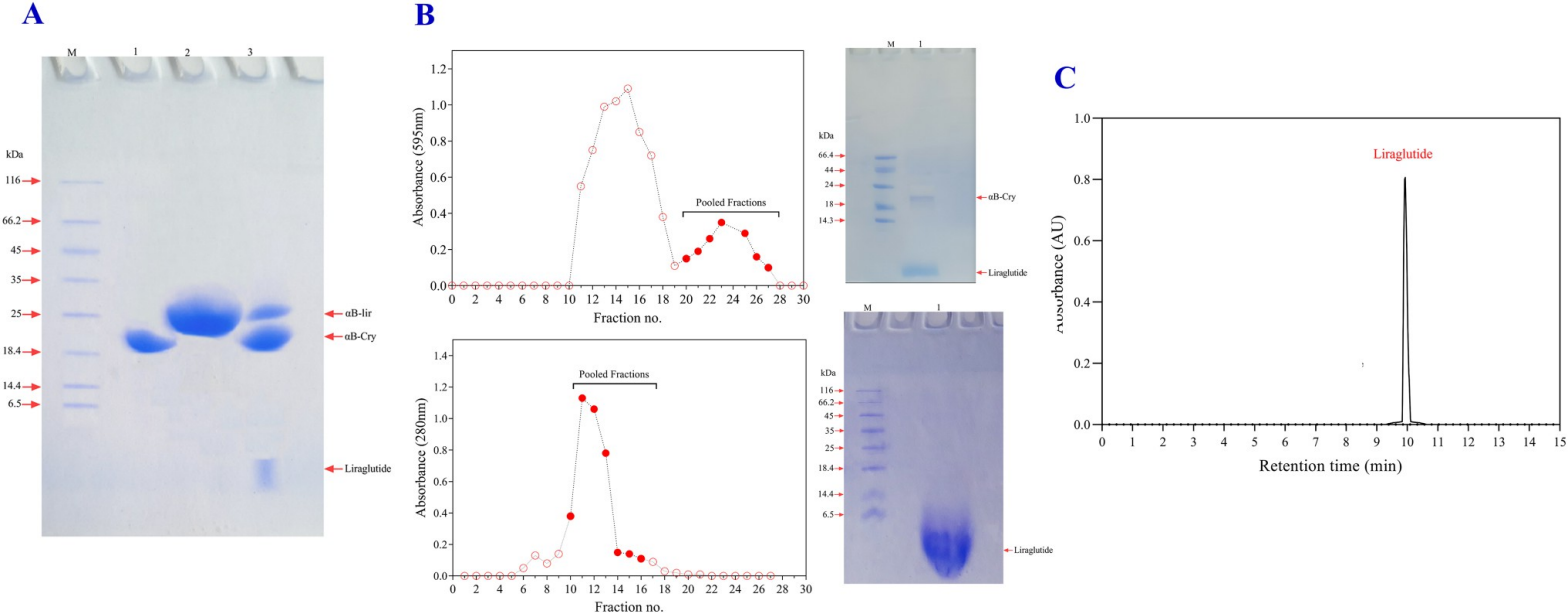
From the specific chemical cleavage of the αB-lir hybrid protein to the purification of LPP. **A**) The CNBr was used for cleaving a specific peptide bond at the boundary methionine between partner protein (human αB-Cry) and LPP. Lanes **1** and **2**, respectively, indicate human αB-Cry and αB-lir, while lane **3** shows αB-lir after the specific chemical cleavage at the boundary methionine. M is the molecular mass marker. **B**) The αB-lir after the chemical cleavage was subjected to a Sephadex G50 gel filtration column. The upper and lower panels show the first and the second round of purification. The pooled fractions rich in the LPP were analyzed by SDS-PAGE (gel 18%). Lane **1** in the upper and lower panels respectively indicates a semi-purified and a highly pure sample of the LPP. **C**) The pure fraction of LPP was then subjected to a reverse-phase (ProntoSIL 200-5-C18, 250 × 4.6 mm) high-performance liquid chromatography column (HPLC) equipped with a UV detector (Smartline 2500 KNAUER). The sample was eluted at a 1 mL/min flow rate with a 0–60% linear gradient of acetonitrile over 15 min at 25°C.

After successfully purifying the hybrid protein (αB-lir), cyanogen bromide was used to separate the LPP from the carrier protein. After 24 hours of incubation in the presence of cyanogen bromide (weight ratio of 1:1), a substantial amount of LPP (3.3 kDa) was released from the hybrid protein. As SDS-PAGE analysis was used to monitor the digestion performance, a minor portion of the intact hybrid protein was also detected (Fig 2A). According to the result of the SDS-PAGE assessment, the digestion efficiency was estimated to be about 70%.

**Fig 3:**
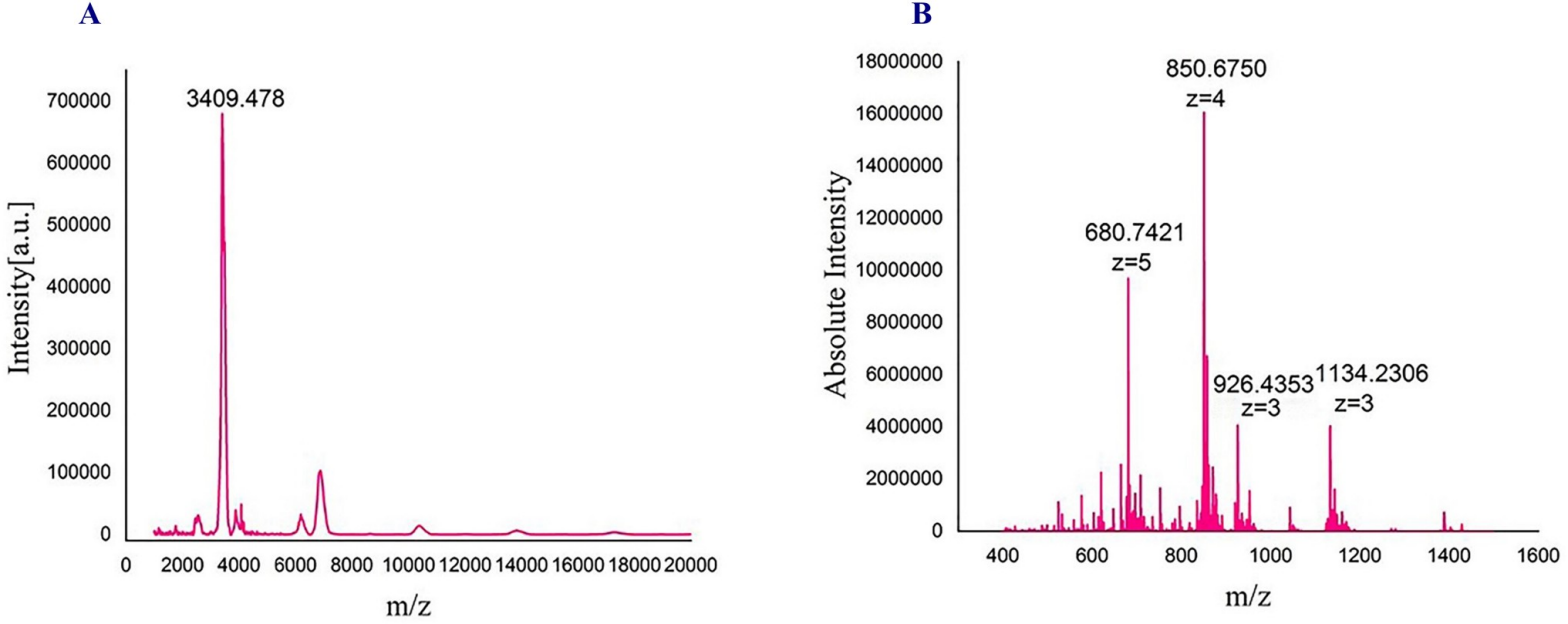
Mass spectroscopy analyses of the LPP. MALDI-TOF mass spectroscopy analysis of the LPP (**A**), and Orbitrap High-resolution Liquid chromatography mass spectrometer analysis of m/z of the peptide (**B**).

In the next step, the purification of the LPP was performed on a gel filtration chromatography using Sephadex G-50 column (0.5 cm×100 cm). The LPP-rich fractions were collected and applied once again to the same column with the aim to get a highly pure peptide sample. As indicated in Fig 2B, the chromatogram of the purification displays that the proteins were eluted from the gel filtration column in two major peaks. According to the results of SDS-PAGE assessment, the first peak is related to the intact hybrid protein (αB-lir) and human αB-Cry, while the second peak mainly contains LPP. As indicated by the SDS-PAGE analysis, a small amount of the carrier protein (αB-Cry) was also detected in the second peak. Therefore, in order to achieve the high purity of this therapeutic peptide, the same gel filtration chromatography was repeated once again. Finally, the efficiency of the method used to purify the LPP was evaluated by both SDS-PAGE and reverse-phase HPLC analyses (Fig 2C). The HPLC analysis suggested the presence of a single peak corresponding to the LPP with a purity which was estimated to be about 98%. Also, approximately 5 mg of a highly pure peptide sample was obtained from one liter of the culture medium. Finally, the mass of the produced LPP was assessed using a Bruker Autoflex Speed MALDI-TOF mass spectrometer (Fig 3A).

The expected mass for the LPP on the basis of the amino acid sequence is 3383.72 Da. However, we obtained peaks corresponding to ~3409 Da. Variation in the observed mass (~26 Da) can be attributed to probable mixing with the matrix and getting conjugated with the peptide. Next, to have clarity about purity and molecular weight, we performed Orbitrap High-resolution liquid chromatography mass spectroscopy (LCMS) analysis of the peptide. We obtained peaks at 680. 94 Da (z = 5), 850.675 Da (z = 4), 1134.23 Da (z = 3), suggesting that the peptide purified by the above-described methods is of high purity and mass obtained matches with the theoretical value (Fig 3B). Furthermore, a peak at 926.43 corresponds to the fragmented mass of the LPP with ions z = 3.

### 3.3. Structural analyses of the produced LPP and αB-lir

**Fig 4:**
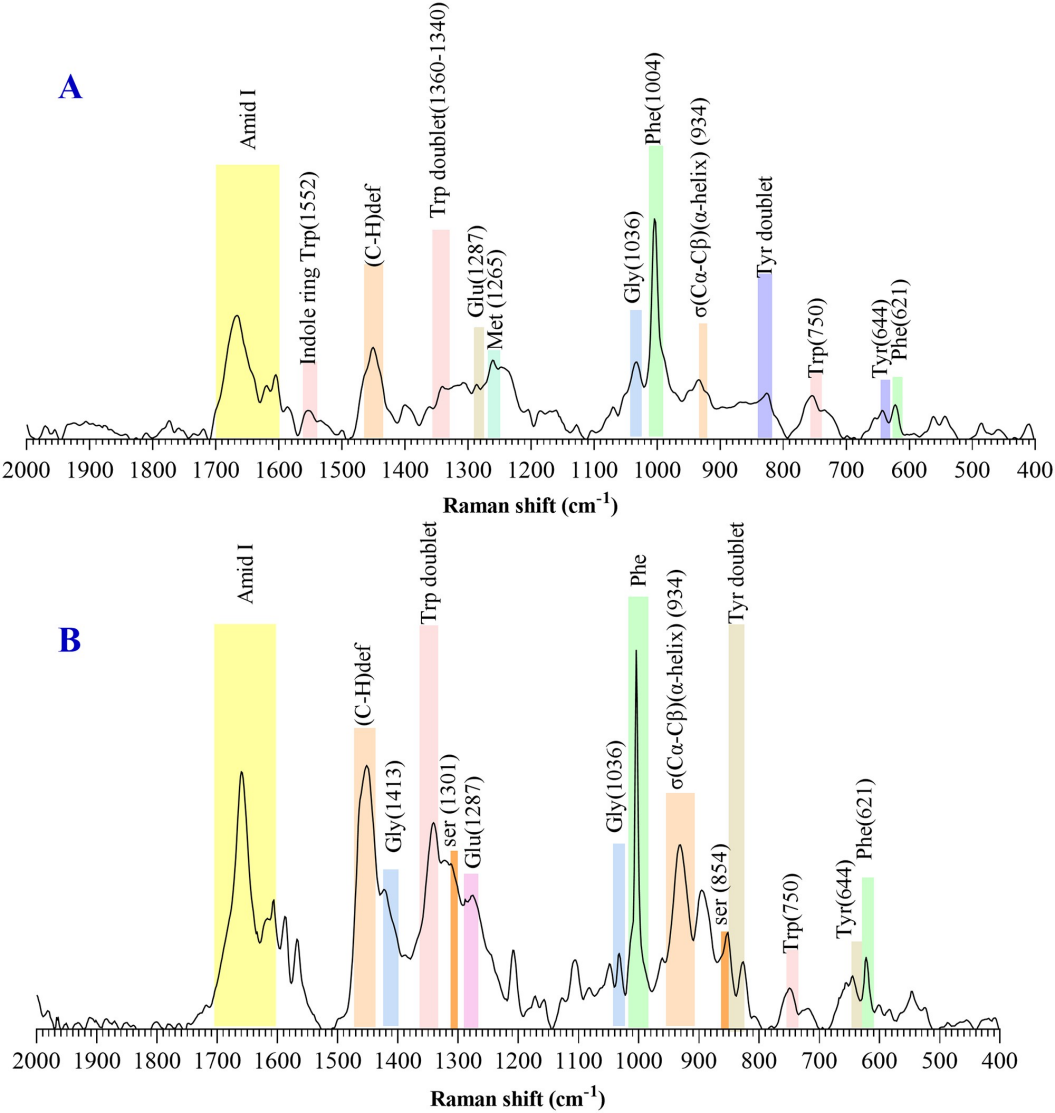
Raman spectral analyses of the αB-lir hybrid protein and LPP in the fingerprint region. The structure of αB-lir and LPP was characterized by Raman spectroscopy in the fingerprint region. Raman spectra of the LPP (**A**) and αB-lir hybrid protein (**B**) were indicated.

In order to study the structure of LPP and αB-lir, the Raman spectra in the area called fingerprint region (1800–400 cm^-1^) were obtained. As shown in Fig 4, the areas belong to the aromatic residues (Phe, Trp, Tyr), and those related to the abundant amino acids are well defined in the Raman spectra of the LPP and αB-lir hybrid protein.

The Fermi-doublet intensity ratio of tryptophan (I_1360_/I_1340_) indicates the environment around the indole ring [52, 53]. This ratio for the hybrid protein and LPP was 0.61 and 0.51, respectively, suggesting the environment around the indole ring is hydrophilic in both LPP and αB-lir hybrid protein. Also, the intensity ratio of the tyrosine doublet (I_850_/I_830_) obtained for the hybrid protein and LPP were 0.8 and 1.39, respectively. Our results suggest that the phenolic OH group of tyrosine residues in both serve as a strong hydrogen bond acceptor and donor.

**Fig 5:**
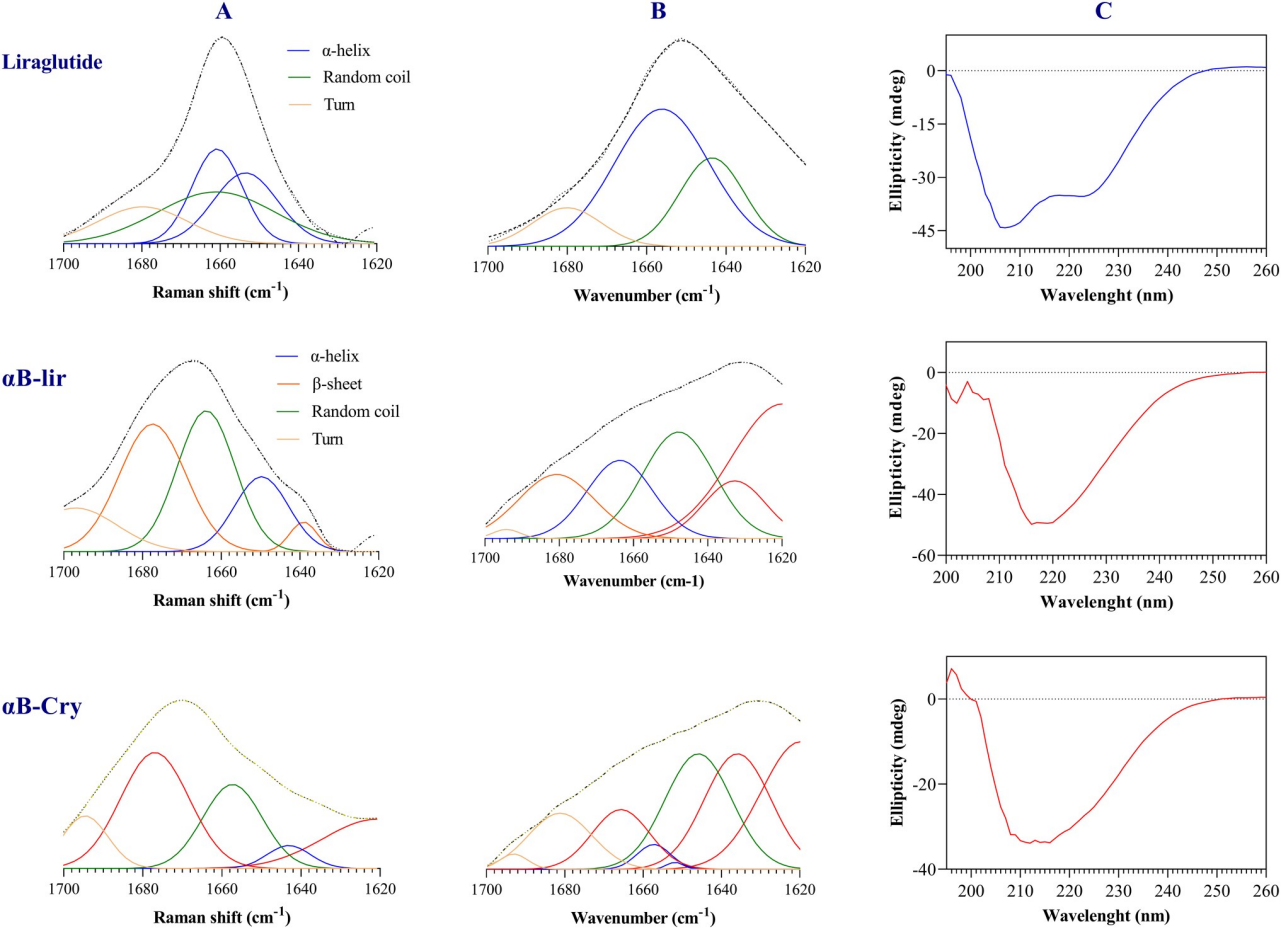
The secondary structure analyses of LPP and αB-lir. For the analyses of the secondary structures, three different methods as Raman (**A**), ATR-FTIR (**B**) and far UV-CD (**C**) were applied. During the Raman and FTIR assessments, the samples were used as powder while the amide band I was deconvoluted by GRAMS (version 9.2) [38]. In the far UV-CD analyses, the LPP was dissolved in phosphate buffer pH 8.1, while αB-lir and αB-Cry were prepared in 100 mM acetate buffer, pH 5.2. In the far UV-CD assessments, the protein/peptide concentration was fixed at 0.2 mg/mL, and the data were deconvoluted by DichroWeb [43]. The upper, middle and lower panels respectively stand for the secondary structure analyses of the LPP, αB-lir and αB-Cry.

Additional to the structural characterization with Raman spectroscopy, information about the secondary structures were obtained by examining the amide band-I in the Raman and FTIR spectra (Fig 5A & 5B). The secondary structure analysis by Raman spectroscopy suggested the LPP as a peptide-rich in α-helix. Further investigations on the secondary structure of the LPP and αB-lir were done by ATR-FTIR (Fig 5B) and far UV-CD (Fig 5C) analyses. All these assessments suggested the LPP as an α-helix rich peptide, which agrees with the previous evaluations [54, 55].

The CD spectrum of the LPP (Fig 5C) shows the presence of two minima at 208 nm and 220 nm, suggesting the dominant presence of the α-helical structure. The percentage of the secondary structures were also calculated and summarized in Table 1. In general, our findings by these three methods confirm that the LPP mostly contains α-helical structure, in agreement with the previous reports [56]. The αB-lir was also compared according to the secondary structural contents with the carrier protein (αB-Cry). The far UV-CD spectra suggest a single minimum at 215 nm for human αB-Cry and a minimum at 217 nm for αB-lir hybrid protein, suggesting the presence of a substantial amount of β-sheet in their structures. Comparison of their CD spectra shows a critical reduction in the molar ellipticity of αB-lir, revealing a significant variation in its secondary structures as compared with human αB-Cry. The two other methods also suggest that similar to human αB-Cry, the αB-lir is a β-sheet rich protein. The results obtained by these three methods suggest that the content of β-sheet was significantly reduced, while the amount of α-helical structure was increased in the hybrid protein when compared to human αB-Cry. The increment of α-helical content in the hybrid protein might also be the result of the attachment of an α-helix rich peptide to the human αB-Cry. Also, the critical reduction in the β-sheet content of αB-lir may reflect the different folding of the hybrid protein after linking to the LPP.

**Table 1:** The protein secondary structure elements (%) of LPP, αB-lir and αB-Cry obtained by three different methods as Raman, ATR-FTIR and far UV-CD.

		α-helix	β-Sheet	Random coil	Turn
**LPP**	Raman	57.2	_	25.8	**17.0**
	FTIR	60.7	_	26.8	**12.5**
	CD	60.5	5.7	20.1	**13.7**
**αB-lir**	Raman	17.1	39.1	34.3	**9.5**
	FTIR	18.4	36.4	27.5	**17.7**
	CD	16.8	39.5	31.3	**12.4**
**αB-Cry**	Raman	2.6	52.4	28.1	**16.9**
	FTIR	3.3	55.9	26.9	**13.9**
	CD	9.7	49.5	27.2	**13.6**

### 3.4. Assessment of the oligomerization status of the αB-lir hybrid protein

**Fig 6:**
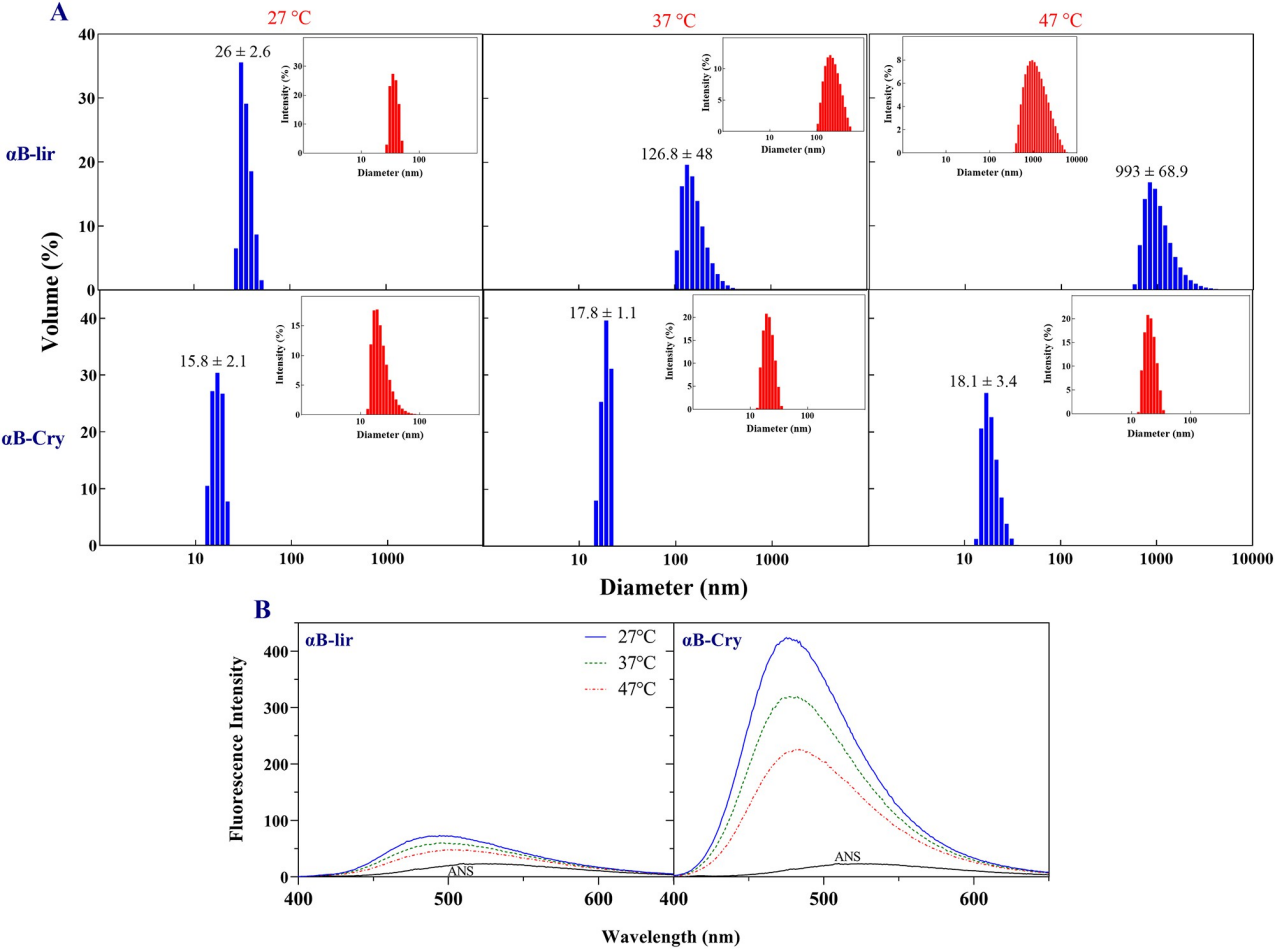
Oligomerization status and surface hydrophobicity of αB-lir hybrid protein. **A**) The hydrodynamic diameters of αB-lir hybrid protein and human αB-Cry (1 mg/mL in phosphate buffer, pH 7.4) were measured at different temperatures by a DLS instrument. **B**) For the ANS fluorescence assessments, the αB-lir and αB-Cry were prepared in 50 mM sodium phosphate buffer, pH 7.4 and the protein concentrations were fixed at 0.15 mg/mL. Then, their surface hydrophobicity analyses were performed at various temperatures in the presence of a fixed ANS concentration (100 μM). The excitation of the protein/ANS complex was done at 365 nm, and the emission spectra were collected between 400–600 nm. Also, the excitation and emission slits were fixed at 5 and 10 nm, respectively [44].

Human αB-Cry has an intrinsic ability to form large oligomers [57]. The hydrodynamic diameter of the oligomers was measured for αB-lir and compared to that of human αB-Cry at different temperatures (Fig 6A). At 27°C, the average oligomeric size of human αB-Cry was 15.8 ± 2.1, while at this temperature αB-lir oligomer indicates a diameter of 26 ± 2.6. With increasing temperature to 47°C, the mean oligomeric size of the carrier protein was increased to 18.1 ± 3.4, but the observed diameter for the hybrid protein was significantly higher (993 ± 68.9). The αB-lir indicated a higher ability to form larger oligomers in all temperatures studied. This feature of the hybrid protein might be highly important in its biological activity and half-life. By creating a large oligomer at the injection site, the releasing process is expected to be slow and stay longer in the bloodstream. The oligomerization status of the hybrid protein was further examined in the solvent used for its purification. Again, the αB-lir hybrid protein indicated larger oligomers than human αB-Cry.

The solvent-exposed hydrophobic surfaces play a significant role in protein oligomerization [44, 58]. Therefore, we used ANS fluorescence assessment with the aim to compare this important feature between human αB-Cry and αB-lir hybrid protein. As indicated in Fig 6B, human αB-Cry shows a more significant ANS fluorescence intensity than the hybrid protein in all temperatures examined, suggesting its higher solvent-exposed hydrophobic surface. The lower hydrophobic surface of the αB-lir can be explained with its ability to the formation of the larger oligomers in which most of the hydrophobic surfaces are hidden in the protein-protein interface interaction areas inside the oligomers.

### 3.5. The *in vivo* biological activity assessments of LPP and αB-lir hybrid protein

**Fig 7:**
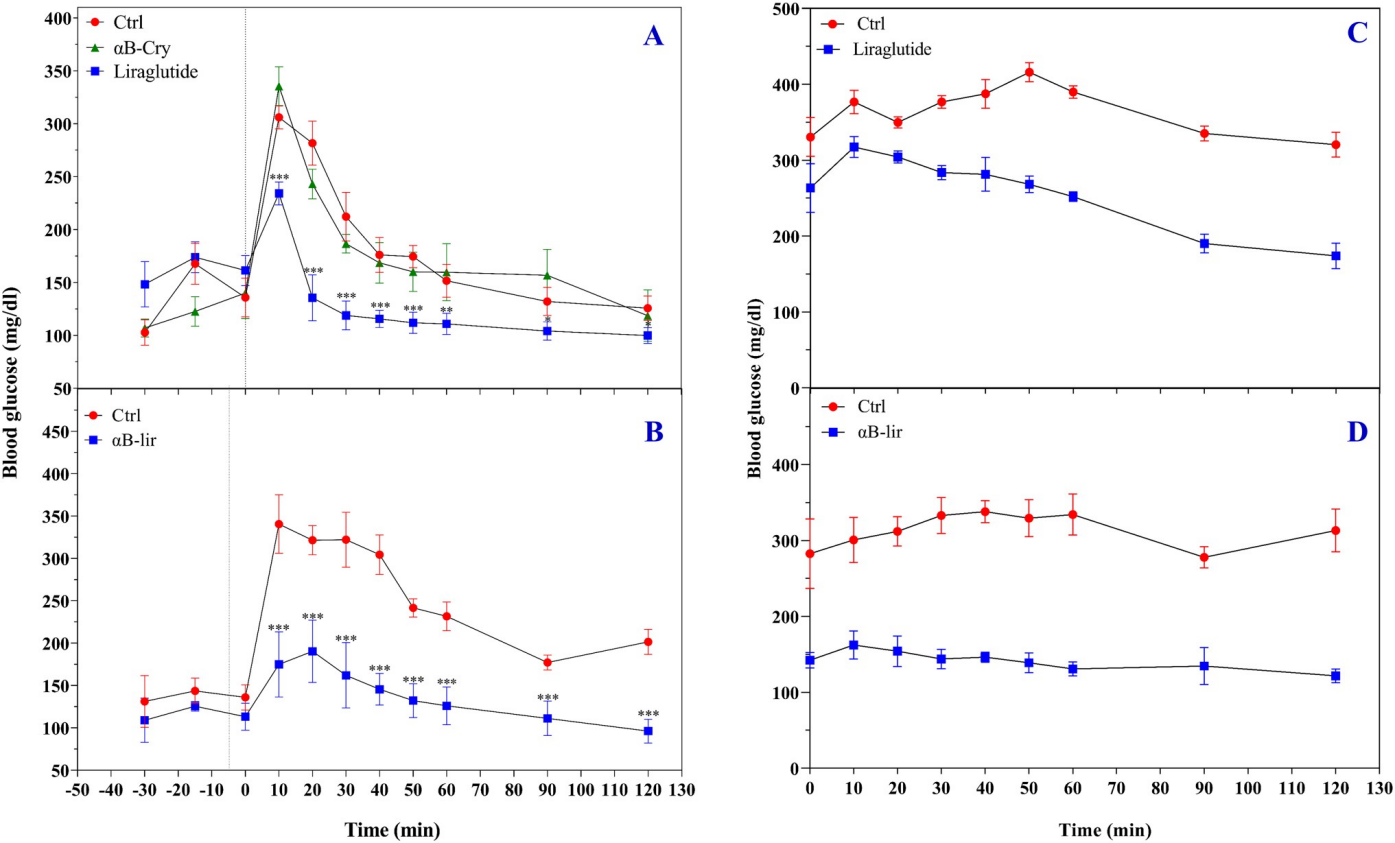
The glucose-lowering activity of LPP and αB-lir. The glucose-lowering effects (IPGTT) of LPP (**A**) and αB-lir (**B**) were studied. A single-dose of the LPP (200 μg/kg body weight) and the αB-lir hybrid protein (1.4 mg/kg body weight) was injected to the mice and the blood glucose concentration was measured during 120 minutes [46]. The concentration of injected glucose was 1.5 mg/g body weight of the normal mice, and the injection was done at time zero. In the case of αB-lir, the blood glucose level was monitored after 24 hours of the initial injection. The blood glucose levels of STZ-induced diabetic mice (n = 6) were also measured after a single-dose, 200 μg/kg and 1.4 mg/kg body weight, respectively, for the injection of the LPP (**C**) and αB-lir (**D**). The data were significantly different from the control group (p<0.05).

Our biological assessments assessed the ability of the produced LPP and αB-lir hybrid protein to reduce blood glucose levels in the healthy mice by an appropriate glucose tolerance assay [46]. The mice were categorized into four groups as A (received LPP), B (control of A), C (received αB-lir) and D (control of C). As shown in Fig 7A, after a single dose glucose injection, the blood sugar levels at its maximum concentration were 230 mg/dL in group A compared to 302 mg/dL in group B. The blood glucose levels in mice belonging to group A reach the normal state faster than those in group B. The glucose-lowering effect of αB-lir hybrid protein was also examined (Fig 7B). A long-acting glucose-lowering response was observed for the αB-lir hybrid protein, but its maximum activity was detected after 24 hours of its initial injection.

The results in Fig 7B suggested that αB-lir is capable of preventing the dramatic increase in the blood glucose levels after injection of the sugar. Also, the difference in the blood glucose levels between the groups C and D remained unchanged throughout the experiment, so that during 90 minutes, the blood glucose levels in the experimental group (C) reached normal state (110 ~ mg/dL), while in the control group (D) the glucose concentration remained at high level (180 ~ mg/dL). These results indicated that the purified LPP and the hybrid protein improve glucose resistance. We also used STZ-induced diabetic mice to evaluate the blood-glucose-lowering response of the LPP and αB-lir hybrid protein (Fig 7C & 7D). As mentioned in the experimental section, a slightly different protocol was used to test their glucose-lowering effect in diabetic mice. The glucose levels in the control mice (group B) were high during the experiment, while in the LPP injected mice (group A), the sugar levels began to decrease and lasted for 10–120 minutes (Fig 7C). The results obtained from the diabetic mice that have been injected with the hybrid protein are shown in Fig 7D. As indicated in this figure, the blood glucose levels in the αB-lir-receiving mice (group C) is almost close to the normal levels compared to the control animals (group D). This difference reflects the slow release of hybrid protein to the bloodstream, which increases the duration of its activity in the body. Therefore, the αB-lir hybrid protein can be considered as a long-acting incretin mimic.

**Fig 8:**
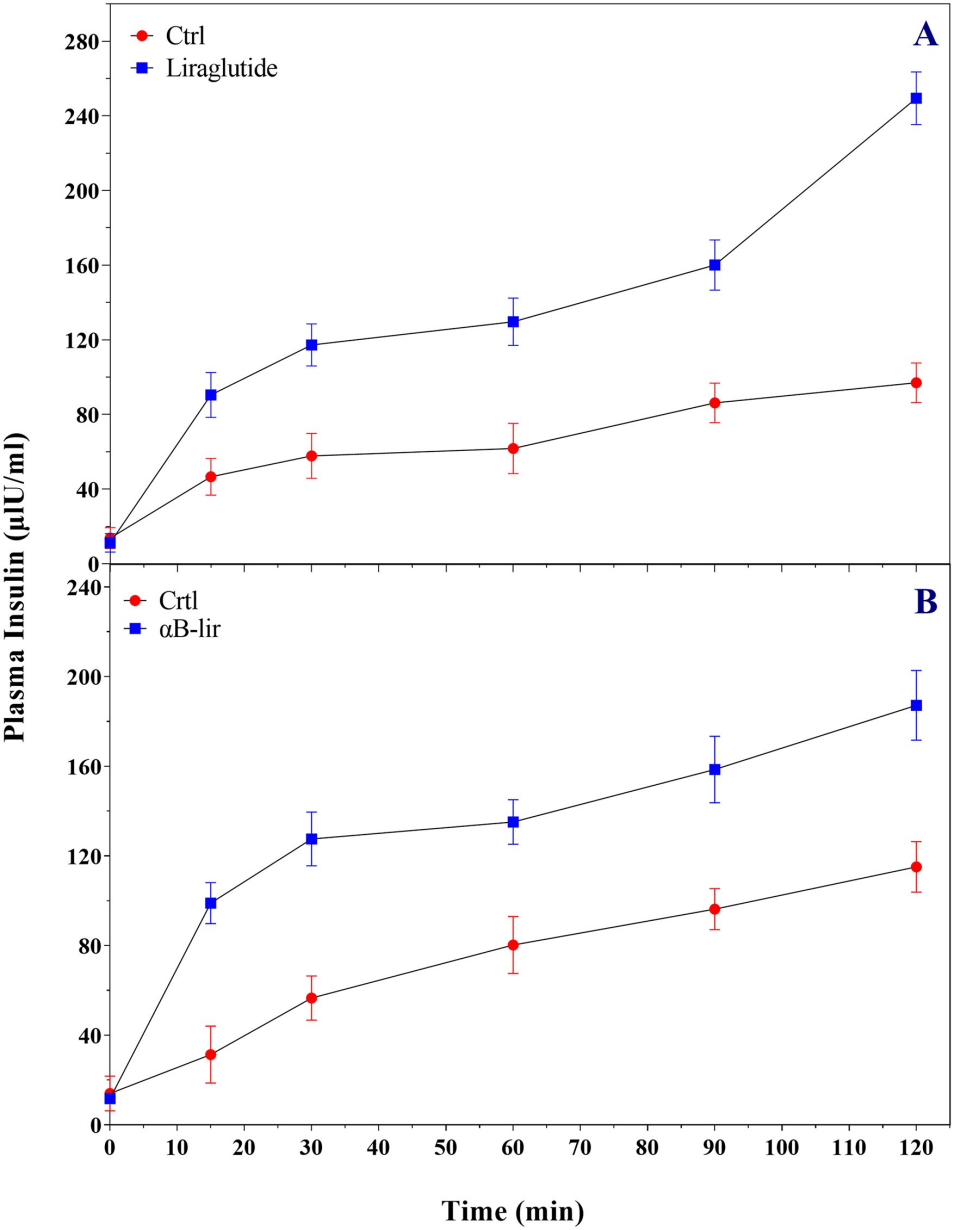
The analyses of the insulin induced secretion of LPP and αB-lir. The effect of LPP (200 μg/kg body weight) (**A**) and αB-lir (1.4 mg/kg body weight) (**B**) on stimulation of the insulin secretion was studied by a single-dose injection. The plasma insulin levels of normal mice (n = 6) were measured by an appropriate insulin ELISA kit [51]. In the case of αB-lir, the plasma insulin level was monitored after the initial injection. The glucose concentration was 1.5 mg/g body weight of normal mice as injected at the time zero. All data were significantly different from the control group (p<0.05).

The stimulation of insulin secretion by LPP and αB-lir was also investigated with the healthy mice (Fig 8).

The appropriate times to assay the insulin secretion were chosen from the glucose-lowering profile of the LPP and αB-lir. The different times in which the protein/peptide indicated glucose-lowering activities were also chosen to test their insulin stimulating secretion. The αB-lir indicated significantly higher insulin stimulating secretion than the LPP. Moreover, in the case of αB-lir, the insulin-induced secretion occurs longer than the LPP, further suggesting the hybrid protein as a long-acting incretin mimic. Overall, our results with the plasma insulin assay were consistent with the observed responses on the blood glucose levels.

## 4. Discussion

The effect of incretin has been indicated to reduce in the patients with type 2 diabetes significantly. Previous clinical studies revealed that GLP-1 is a vital incretin hormone, playing a key role in the pathophysiology of diabetes mellitus [59]. The main challenge in using GLP-1 as an anti-diabetic medicine is its short half-life, mostly related to its enzyme inactivation with DPP-IV and its fast renal clearance [60]. Therefore, researchers have always sought to design various DPP-IV-resistant incretin mimics either by developing their conjugated forms or applying DPP-IV inhibitors. The main goal in the design is to create incretin mimics with a longer half-life than wild-type GLP-1 that can continuously activate GLP-1 receptors for a longer period [61]. The GLP-1 analogues are divided into long-acting and short-acting based on their pharmacokinetic properties [14]. The design of long-acting analogues is done so that in addition to resisting enzymatic degradation, their renal removal is largely prevented and their release into the bloodstream occurs over a long period.

In this study, we proposed a novel way to produce the LPP through the formation of a hybrid protein with human αB-Cry (Fig 1A). This carrier protein protects the peptide from fast degradation. In addition, the results of previous studies indicated that the carrier protein (human αB-Cry) alone is highly expressed when a bacterial host system was used [62]. So by linking this anti-diabetic peptide to αB-Cry chaperone, the expression of the peptide also increases (Fig 1B). Then, using an innovative and easy to run method, many impurities associated with the hybrid protein were removed by precipitation. Finally, we used ion-exchange chromatography to achieve a highly pure sample of the hybrid protein. Since there is only one methionine at the end of the carrier protein, the intact LPP can be released by chemical cleavage in the presence of CNBr. As shown in Fig 2A, the efficiency of the chemical cleavage was estimated to be 70% which is acceptable. The intrinsic ability of the carrier protein (αB-Cry) and the hybrid protein for appearing in the large oligomer (Fig 6) provide an important advantage for the easier purification of the LPP by size exclusion chromatography which can be scaled up to the industrial level. This feature finally helps the drug peptide to be easily and efficiently purified from the carrier protein or a small portion of the undigested hybrid protein (Fig 2C). Finally, the LPP was purified from the other impurities at the high level of purification (Fig 2C). The mass spectroscopic analysis also clearly confirmed the exact mass of the drug peptide obtained in this study (Fig 3). The result of mass spectroscopy analysis suggested that the peptide has been precisely released at the end of the carrier protein and also remains unchanged during expression and the downstream purification steps.

The secondary structures have been used as an essential index to characterize proteins and peptides [63]. Previous studies suggested an α-helical rich structure for LPP [37, 40, 64]. Therefore in the current study, Raman, ATR-FTIR, and CD spectroscopy analyses were used to carefully investigate the secondary structures of the produced LPP and the hybrid protein. All three methods showed well that the LPP produced in our study was rich in α-helical structure. Also, the previous studies have introduced human αB-Cry as a protein with high contains of β-sheet structure [65]. The spectroscopic assessments performed in this study also suggested that the β-sheet was the predominant structure in the hybrid protein (Fig 5). The secondary structural estimates indicated that in comparison to human αB-Cry, β-sheet and helical structures are respectively decreased and increased in the hybrid protein. The structural transition at the secondary structures suggested that hybrid protein may have a different folding than the carrier protein (human αB-Cry).

On the other hand, an increase of the α-helical content in the hybrid protein can also be related to the attachment of the LPP as a helix-rich peptide to the carrier protein. As reported already, the α-helical structure of liraglutide plays an important role in its proper interaction with the membrane receptor [62], so preserving the helical structure of the therapeutic peptide after linking to the carrier protein plays an important role in its increting activity as indicated in Figs 7 & 8. In this study, blood glucose-lowering activity in healthy and diabetic mice and the incretin stimulation of insulin secretion were investigated (Figs 7 & 8). The *in vivo* activity analyses suggested that in addition to the LPP, the hybrid protein was also efficiently capable of reducing blood sugar levels and significantly enhancing insulin secretion after their injection into the mice.

Compared to the LPP, the hybrid protein is more active for a relatively long period, leading to a further reduction in the blood sugar levels and a higher stimulation of the insulin secretion by the pancreatic β-cells. The more extended activity of the hybrid protein is partly because linking the carrier protein to the N-terminus of the LPP protects the attached therapeutic peptide from DPP-IV digestion. In other words, it is possible that the fusion protein can adopt a conformation so that the protease-sensitive peptide bond in the peptide moiety is hidden from the enzymatic degradation. A similar phenomenon occurs in liraglutide when attached to its fatty acid [66]. In addition, the attachment of the small peptide (LPP) to a large protein such as human αB-Cry prevents its fast renal filtration. Our DLS assessments suggested that the hybrid protein has an important ability to form large oligomers. Also, ANS binding analysis of the hybrid protein shows that due to the formation of the larger oligomers, most of the hydrophobic surfaces are hidden in the protein-protein interface of the interaction areas inside the oligomers. So, it is possible that after subcutaneous injection, its large oligomers cause the release of active monomers over an extended period.

In summary, although clinical data indicated that liraglutide has better glycaemic efficacy than exenatide [67], our hybrid protein may be a better drug candidate than the liraglutide medicine because it acts over a longer period of time and also lowers blood sugar levels to the greater extent (Fig 7). In addition, activity liraglutide medicine over time in the bloodstream depends on its interaction with human serum albumin. The results of previous research strongly suggested that in diabetic individuals, albumin is extensively glycated. Under this condition, the general carrier of the bloodstream loses its ability to interact with liraglutide via the fatty acid moiety [28]. Therefore, liraglutide may not be an effective therapeutic medicine for diabetic patients who suffer from chronic and high blood sugar levels that show extensive glycation of human albumin [28]. So far, there has been no report of αB-Cry specific binding/interaction to human serum albumin. Hence, the hybrid protein acts independently of this general carrier of the bloodstream, and the glycation of albumin in diabetic patients does not affect its activity. Therefore, our hybrid protein with excellent incretin activity over a more extended period can be considered a novel drug candidate for the future clinical treatment of diabetic patients. Finally, there is no report in the literature on the development of allergy against human αB-Cry. Thus, the carrier protein can be considered a safe molecular partner in the structure of the αB-lir incretin mimic.

Briefly, the major steps of the method used in this study to produce human proinsulin and its important findings are shown (Scheme 1).

**Scheme 1. The important steps of the current study and our major findings are summarized in this scheme**.

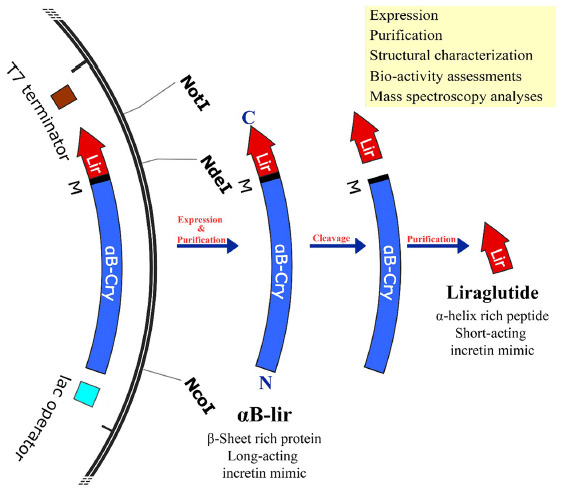



## 5. Conclusion

In this study, a gene structure (αB-lir) was created that had a relatively high expression in the bacterial host system. After successful expression and purification of the hybrid protein, the LPP was released chemically and then purified at high purity. The obtained peptide exhibited an α-helical rich structure, while the mass spectroscopy analysis also confirmed its exact mass. The LPP and the αB-lir hybrid protein revealed important bioactivities, including reducing blood sugar levels and stimulating insulin secretion. The αB-lir was also exhibited more effective bioactivities over a relatively long period than the LPP, which can be attributed to its ability in forming high molecular weight oligomers, releasing slowly into the blood as the active monomers after the subcutaneous injection. The proposed expression/purification method can be applied for the large-scale and industrial production of the LPP due to the use of inexpensive materials and lack of complexity. Also, the hybrid protein whose biological activity is independent of the interaction with human serum albumin is a more suitable alternative than liraglutide for the use in the treatment of patients with severe diabetes where the interaction between albumin and this drug peptide is severely impaired. The results of the current study also introduced a new incretin mimic with more important bioactivities compared to the liraglutide for the possible application in the treatment of diabetes mellitus.

## Supporting information

S1 Fig The primary structure (amino acid sequence) of the liraglutide precursor peptide (LPP) is shown in this figure. (DOCX)

S1 Raw images (PDF)
