# Supplementary material for: A novel strategy for production of liraglutide precursor peptide and development of a new long-acting incretin mimic
Source: PLoS One. 2022 May 2;17(5):e0266833. doi: 10.1371/journal.pone.0266833 (PMC9060347; doi:10.1371/journal.pone.0266833)
Supplement: S1 Fig — (DOCX) [file pone.0266833.s001.docx]

**Supporting information**

**A novel strategy ‌for production of liraglutide precursor peptide and development of a new long-acting incretin mimic**

Samaneh Ahmadi^1^, Mohammad Bagher Shahsavani^1^, Zohreh Tavaf^1^, Rawayh Muslim Albaghlany^1^, Ashutosh Kumar^2^, Ali Akbar Moosavi-Movahedi^3^, Reza Yousefi*^1,3^


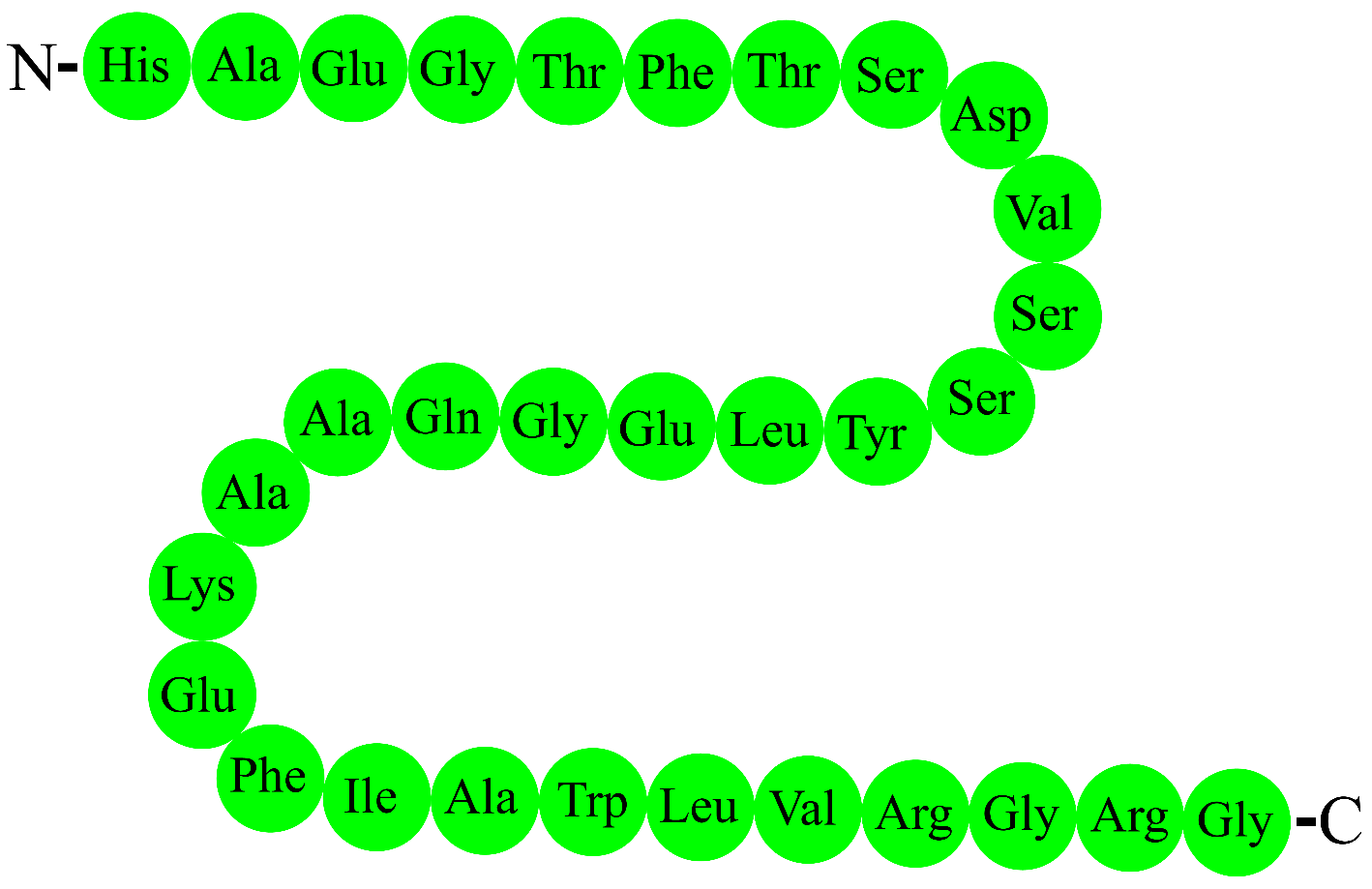


Fig. S1. The primary structure (amino acid sequence) of the liraglutide precursor peptide (LPP) is shown in this figure.
