# Supplementary material for: A novel strategy for production of liraglutide precursor peptide and development of a new long-acting incretin mimic
Source: PLoS One. 2022 May 2;17(5):e0266833. doi: 10.1371/journal.pone.0266833 (PMC9060347; doi:10.1371/journal.pone.0266833)
Supplement: S1 Raw images — (PDF) [file pone.0266833.s002.pdf]

Fig. 1B

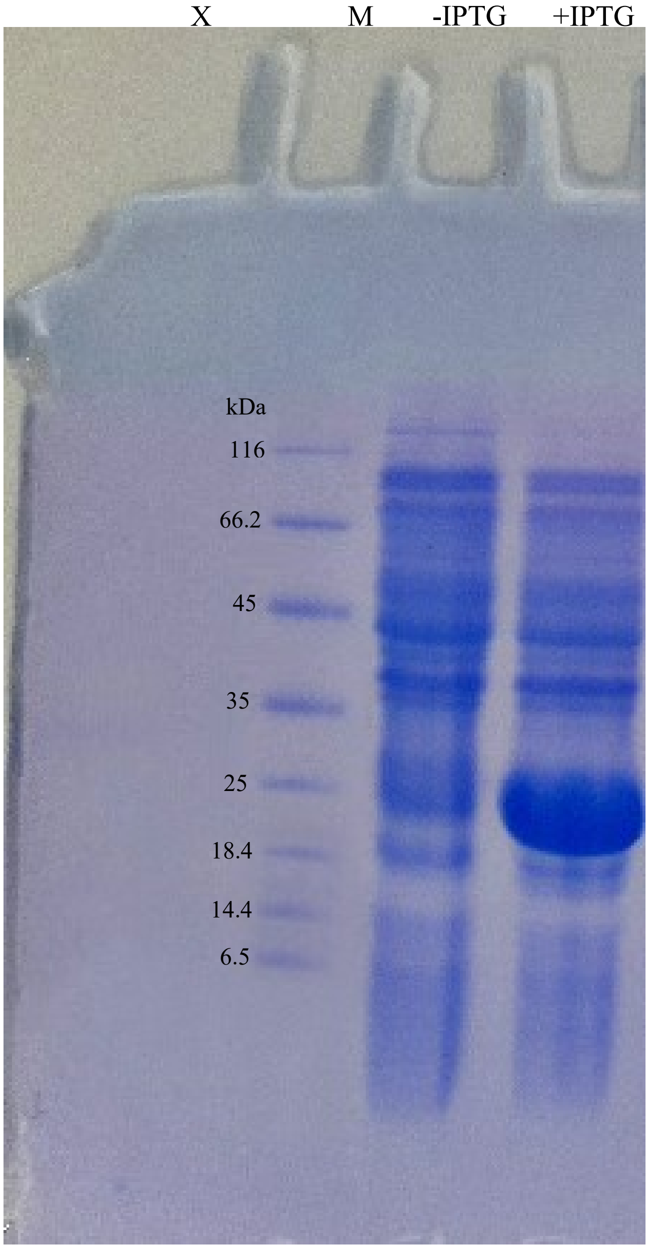

Fig.1B. The expression of αB-lir hybrid protein. The expression of αB-lir hybrid protein was assessed by SDS-PAGE analysis (gel 12%). The αB-lir expression in the absence and presence of 0.25 mM IPTG is indicated, and M shows the protein mass markers**.**

Fig. 1C

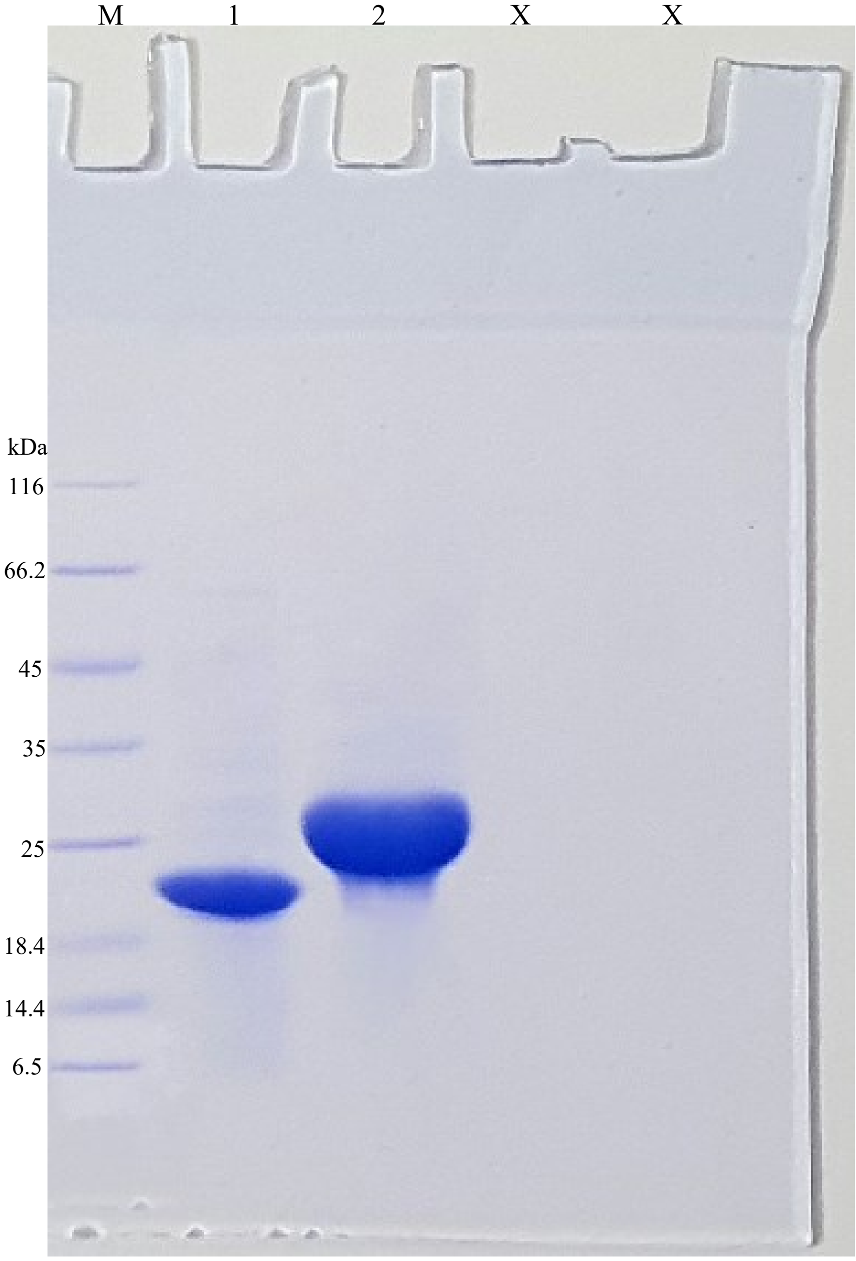

Fig. 1C. The purification of αB-lir hybrid protein. The αB-lir was purified using the precipitation method followed by a DEAE column. Then, the pure αB-lir was analyzed on a reducing SDS-PAGE (gel 12%). Lanes **1** and **2** stand for human αB-Cry and αB-lir hybrid protein, respectively. Also, **M** indicated the protein mass marker.

Fig. 2A

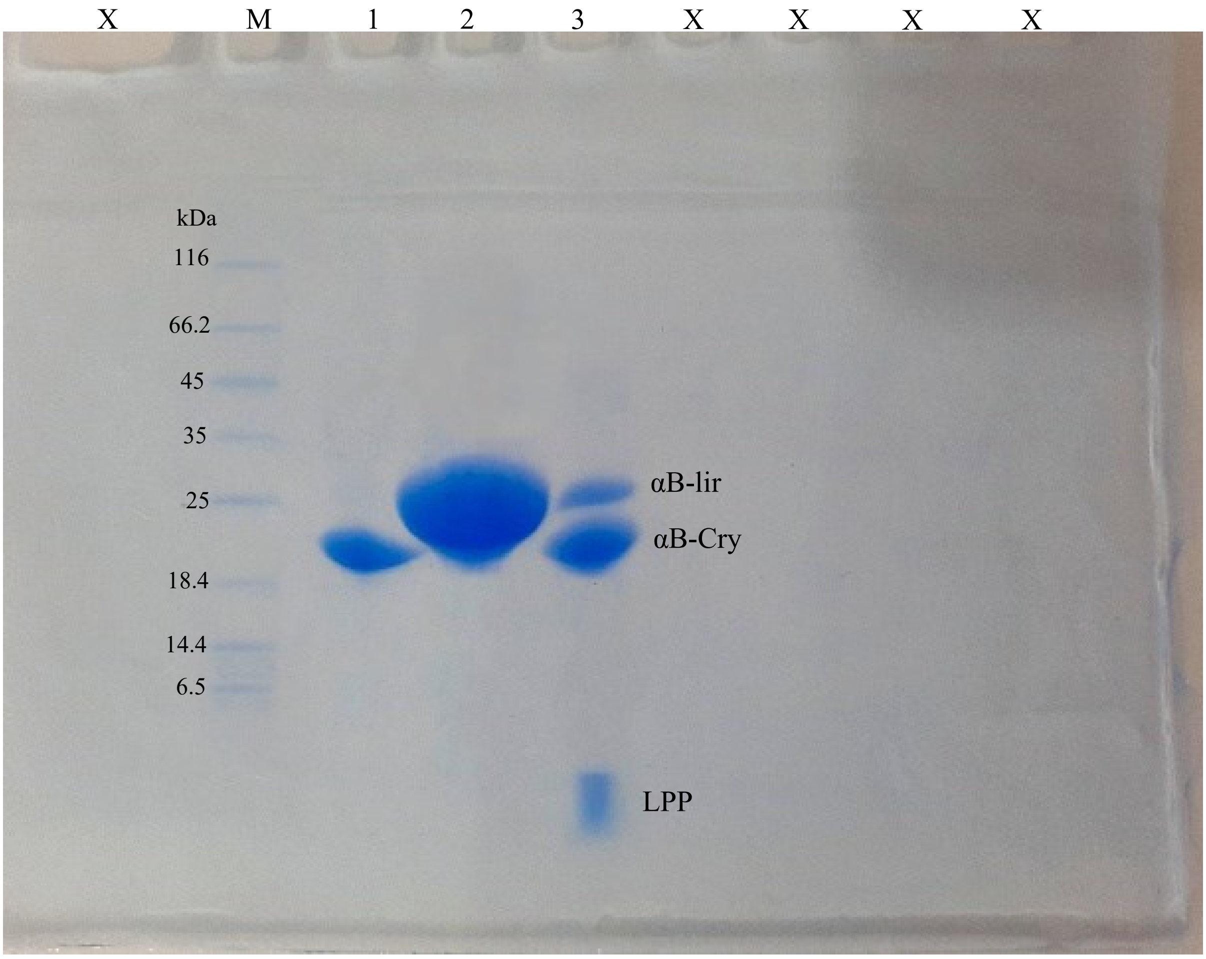

Fig.2A. The specific chemical cleavage of the αB-lir hybrid protein. The CNBr was used for cleaving a specific peptide bond at the boundary methionine between partner protein (human αB-Cry) and LPP. Lanes **1** and **2**, respectively, indicate human αB-Cry and αB-lir, while lane **3** shows αB-lir after the specific chemical cleavage at the boundary methionine. **M** is the molecular mass marker.

Fig. 2B

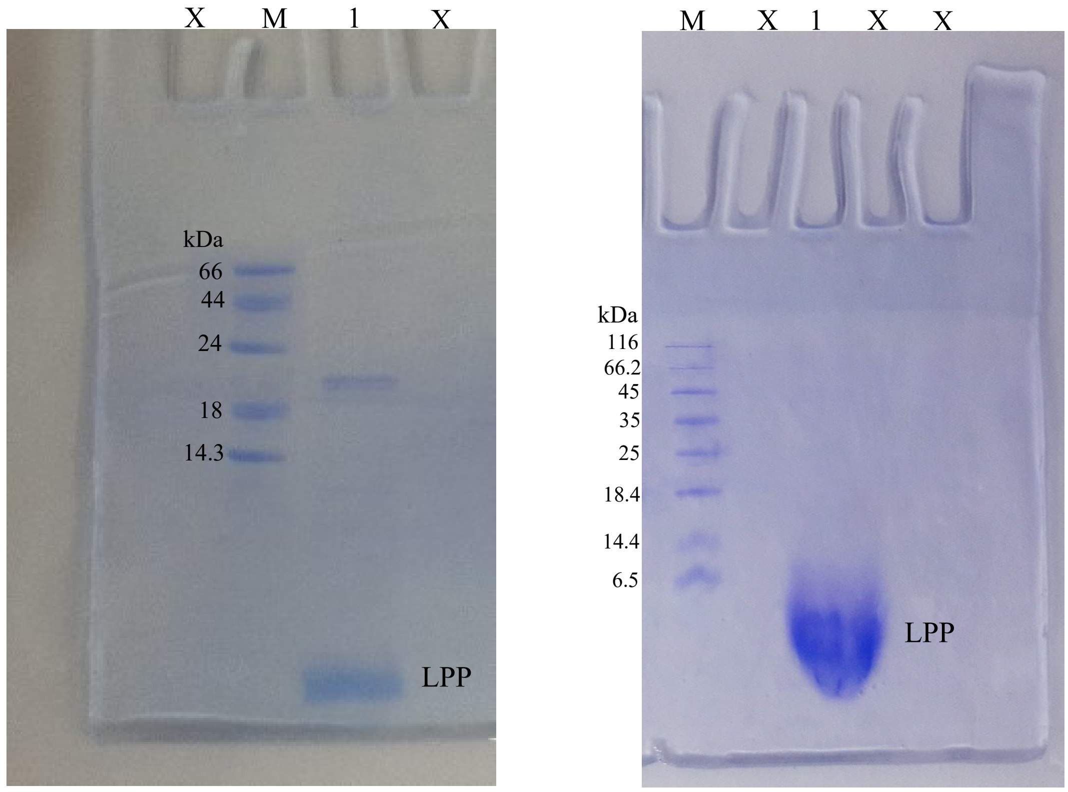

Fig.2B. Purification of the LPP. The αB-lir after the chemical cleavage was subjected to a Sephadex G50 gel filtration column. The pooled fractions rich in the LPP were analyzed by SDS-PAGE (gel 18%). The left and right gels show the first and the second round of purification. Lane **1** in the left and right gels respectively indicates a semi-purified and a highly pure sample of the LPP.

All SDS-PAGE images were taken by phone camera.
